# The complete chloroplast genome sequence of the Tibetan herb *Phlomoides rotata* (Benth. ex Hook.f.) Mathiesen (Lamiaceae)

**DOI:** 10.1080/23802359.2021.1993459

**Published:** 2021-10-20

**Authors:** Yangzom Pema, Chao Ma, Mary Ann C. Bautista, Tao Chen

**Affiliations:** a Tibet Plateau Institute of Biology, Lhasa, Tibet, China; b Fairy Lake Botanical Garden, CAS, Shenzhen, Guangdong, China; c University of Chinese Academy of Sciences, Beijing, China

**Keywords:** *Phlomoides rotata*, lamiaceae, chloroplast genome, phylogeny

## Abstract

*Phlomoides rotata* (Benth. ex Hook.f.) Mathiesen is a perennial herb endemic to Qinghai-Tibet Plateau with important medicinal properties. Here, we sequenced and analyzed the complete chloroplast (cp) genome of *P. rotata* and reconstructed the phylogeny of *P. rotata* based on 24 cp genomes. The genome of *P. rotata* is 151,825 bp in length, including a large single-copy (LSC) region of 83,129 bp and a small single-copy (SSC) region of 17,398 bp. A total of 131 genes were identified, of which 86 are protein-coding genes, 37 are transfer RNA genes, and eight are ribosomal RNA genes. Phylogenetic analyses revealed that the species *P. rotata* is closely related to *Phlomoides alpina* with bootstrap support (BS) values of 100%. Overall, the genomic resources presented in this study will be beneficial for further studies on evolutionary patterns of *P. rotata* and its closely related species.

*Phlomoides rotata* (Benth. ex Hook.f) Mathiesen *(P. rotata)*, a perennial herb belonging to the family Lamiaceae, is endemic to the Qinghai-Tibet Plateau and widely distributes in 2700–4900 m weathered alpine alluvial fans, stony alpine meadows, and floodplain*s* (Wu and Li 1977). As a traditional Tibetan medicinal herb, it is used to treat the traumatic injury, promote blood circulation, and alleviate pain (Liu et al. 2006, Li et al. 2008). However, its classification is currently highly controversial. In this study, the complete chloroplast (cp) genome of this species was sequenced and annotated, which might be useful as a genetic resource for plant identification and further phylogenetic study.

Total genomic DNA was extracted from *P. rotata* leaves collected from Lhasa, Tibet, China (N29°46’41ʺ, E91°10’19ʺ) using the modified CTAB method (Murray and Thompson 1980). The voucher specimen (2020MaC24) was deposited in the Herbarium of Tibet Plateau Institute of Biology (Chao Ma, wvoo@163.com). Paired-end (150 bp) sequencing was conducted in Illumina HiSeq 2500 platform, and the cp reads were filtered using NGS-QC toolkit (Patel and Jain, 2012) and were assembled by using the program SPAdes 3.11.0 (Bankevich et al. 2012) with a k-mer set of 93, 105, 117, and 121. Genome annotation was performed by Plann v1.1.2 (Huang and Cronk 2015), Aragorn v1.2.38 (Laslett and Canback 2004) and Blast v2.6.0. *Phlomoides betonicoides* (MN617020.1) cp genome sequence was used as the reference sequence.

The complete cp genome of *P. rotata* (MZ150795.1) is a typical quadripartite structure of 151,825 bp, consisting of a large single-copy (LSC) region of 83,129 bp, a small single-copy (SSC) region of 17,398 bp, and a pair of inverted repeats (IR) region of 25,649 bp. The assembled cp genome has 131 annotated genes, including 86 protein-coding genes, 37 tRNA genes, and 8 rRNA genes. Among those, 18 are duplicated in IR regions, including seven protein-coding genes, seven tRNA genes, and four rRNA genes. In addition, there are 15 genes with single intron (*trn*K-UUU*, rps*16*, trn*G-UCC*, atp*F*, rpo*C1*, trn*L-UAA*, trn*V-UAC*, pet*B*, pet*D*, rpl*16*, rpl*2*, ndh*B*, trn*I-GAU*, trn*A-UGC*, ndh*A) and 2 genes (*clp*P and *ycf*3) with two introns. The base composition of the complete cp genome sequence was analyzed and found to be 38.46% GC content. Furthermore, the GC content of IR regions was 43.40%, which is higher than that of the LSC (36.65%)/SSC (32.53%).

To investigate its phylogenetic placement in the family, a total of 23 complete cp genomes including one outgroup (*Brandisia swinglei*) were downloaded from NCBI GenBank and aligned with the cp genome of *P. rotata* by using MAFFT (Katoh and Stanley 2013). The maximum likelihood (ML) analysis using GTR-I-G model was performed in RaxML v8.1.11 (Stamatakis, 2014). ML consensus tree revealed that *P. rotata* occupies the basal position in the *Phlomoides* clade (BS = 100%, Figure 1). It diverged earlier than the rest of the *Phlomoides* but it has a higher affinity to *Phlomoides alpina*. The complete cp genome sequence of *P. rotata* is expected to provide new insights into the evolutionary history of *Lamiaceae* and its siblings.

**Figure 1.**
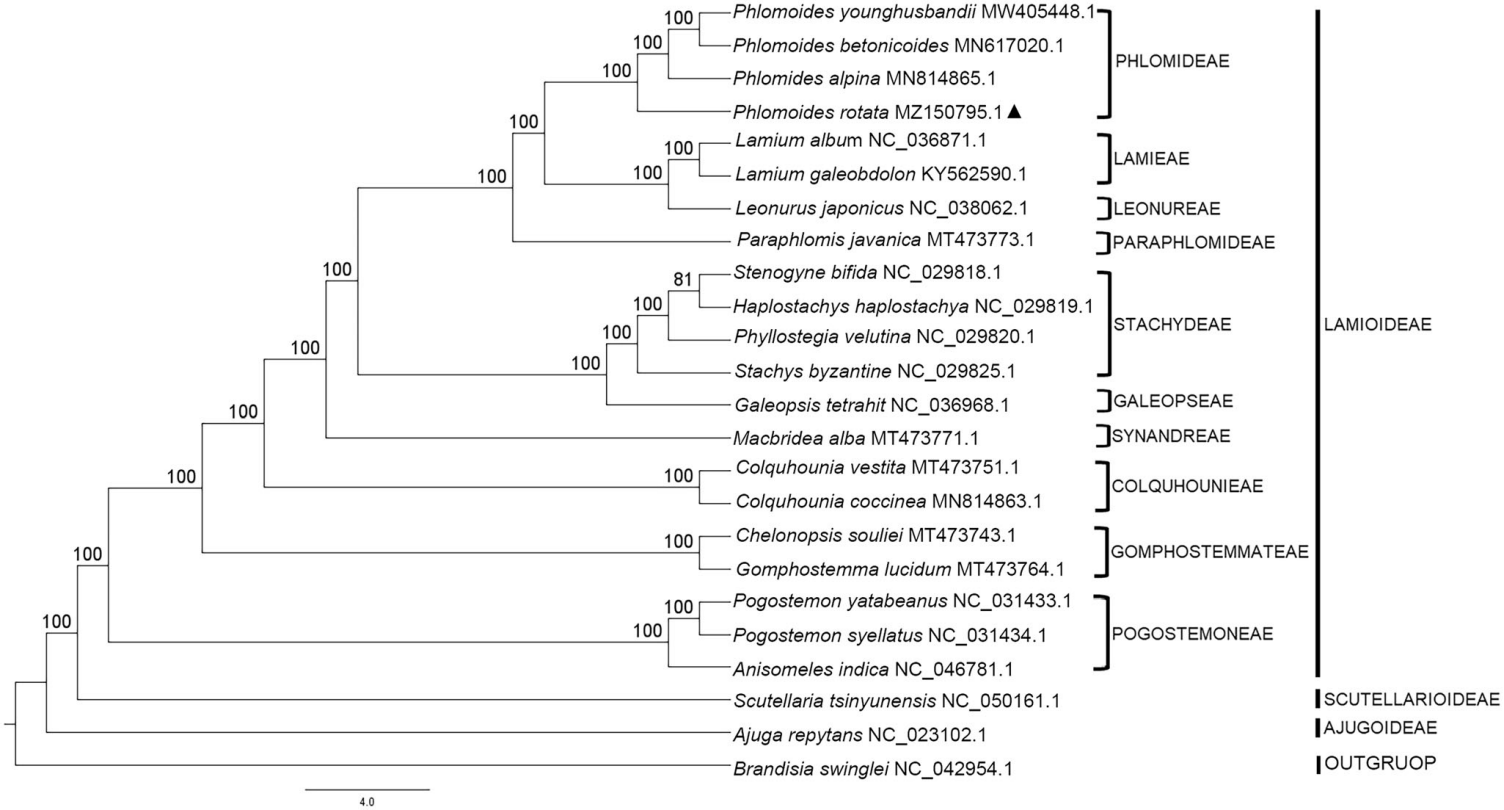
Maximum likelihood (ML) phylogenetic tree based on 24 complete chloroplast genome sequences. Numbers above the nodes represent the bootstrap support. *Brandisia swinglei* was selected as an outgroup.

## Data Availability

The genome sequence data that support the findings of this study are openly available in GenBank of NCBI at (https://www.ncbi.nlm.nih.gov/nuccore/MZ150795.1) under the accession no. MZ150795.1. The associated BioProject, Bio-Sample, and SRA numbers are PRJNA727957, SAMN19066089 and SRR14470730, respectively.
